# Bis(4-pyridylmeth­yl) hexa­nedioate

**DOI:** 10.1107/S1600536808014414

**Published:** 2008-05-17

**Authors:** Jin-Hui Yang, Jian-Min Zhang, Yan-Xue Chen, Jian-Zhi Diao, Zheng Peng

**Affiliations:** a School of Materials Science and Engineering, Shijiazhuang Railway Institute, Shijiazhuang 050043, People’s Republic of China; b School of Chemical Engineering and Technology, Tianjin University, Tianjin 300072, People’s Republic of China

## Abstract

The asymmetric unit of the title compound, C_18_H_20_N_2_O_4_, contains one half-mol­ecule. The mol­ecule lies on an inversion centre and is roughly planar, the chains between the two pyridine rings being only slightly twisted, with torsion angles ranging from 170.9 (1) to 177.2 (1)°. Weak C—H⋯O hydrogen bonds result in the formation of a three-dimensional network.

## Related literature

For related literature, see: Banfi *et al.* (2002 ▶); Magden & Basel (1984 ▶).
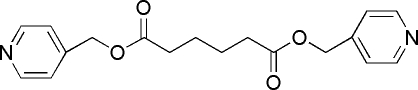


## Experimental

### 

#### Crystal data

C_18_H_20_N_2_O_4_
*M* *_r_* = 328.36
Monoclinic, 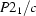

*a* = 9.1489 (18) Å
*b* = 10.164 (2) Å
*c* = 8.9206 (18) Å
β = 102.11 (3)°
*V* = 811.1 (3) Å^3^
*Z* = 2
Mo *K*α radiation
μ = 0.10 mm^−1^
*T* = 113 (2) K
0.12 × 0.10 × 0.08 mm

#### Data collection

Rigaku Saturn diffractometer
Absorption correction: multi-scan (*CrystalClear*; Rigaku, 2005 ▶) *T*_min_ = 0.979, *T*_max_ = 0.988
9823 measured reflections
1918 independent reflections
1288 reflections with *I* > 2σ(*I*)
*R*_int_ = 0.060

#### Refinement

*R*[*F* ^2^ > 2σ(*F* ^2^)] = 0.041
*wR*(*F* ^2^) = 0.106
*S* = 0.98
1918 reflections
109 parameters
H-atom parameters constrained
Δρ_max_ = 0.34 e Å^−3^
Δρ_min_ = −0.24 e Å^−3^

### 

Data collection: *CrystalClear* (Rigaku, 2005 ▶); cell refinement: *CrystalClear*; data reduction: *CrystalClear*; program(s) used to solve structure: *SHELXS97* (Sheldrick, 2008 ▶); program(s) used to refine structure: *SHELXL97* (Sheldrick, 2008 ▶); molecular graphics: *ORTEPIII* (Burnett & Johnson, 1996 ▶) and *ORTEP-3 for Windows* (Farrugia, 1997 ▶); software used to prepare material for publication: *SHELXL97*.

## Supplementary Material

Crystal structure: contains datablocks I, global. DOI: 10.1107/S1600536808014414/dn2349sup1.cif

Structure factors: contains datablocks I. DOI: 10.1107/S1600536808014414/dn2349Isup2.hkl

Additional supplementary materials:  crystallographic information; 3D view; checkCIF report

## Figures and Tables

**Table 1:** Hydrogen-bond geometry (Å, °)

*D*—H⋯*A*	*D*—H	H⋯*A*	*D*⋯*A*	*D*—H⋯*A*
C6—H6*B*⋯O2^i^	0.97	2.56	3.3333 (17)	137

Symmetry code: (i) 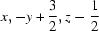.

**Fig. 1.**
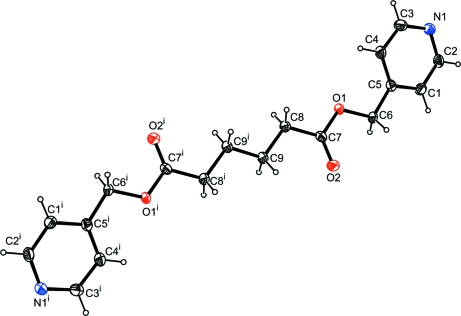
A view of the molecular structure of (I)with the atoms labelling scheme. Displacement ellopsoids are drawn at the 50% probability level and H atoms are shown as small spheres of arbitrary radii. [Symmetry code: (i) 1-x, 1-y, 1-z ]

## supplementary crystallographic information

### Comment

Hexanedioic acid dipyridin-4-ylmethyl ester is a very important intermediate in the synthesis of cephalosporins (Magden & Basel, 1984). Also, it can be used as a ligand designed for the self-assembly of coordination frameworks and architectures (Banfi *et al.*, 2002);

The title compound is arranged around an inversion centre located in the middle of the C9-C9^i^ bond [symmetry code:(i) 1-*x*, 1-*y*, 1-*z* ] (Fig. 1). The molecule is roughly planar, the chains between the two pyridyl rings being only slightly twisted with torsion angles ranging from 170.9 (1) to 177.2 (1)° .

Weak intermolecular C—H···O hydrogen bonds (Table 1) result in the formation of a three dimensionnal network

### Experimental

4-pyridinemethanol (9.82 g, 0.09 mol) and dimethyl adipate (5.22 g, 0.03 mol) were stirred with 200 ml n-octane at 343 k,then titaniun tetraisopropoxide (0.2 g) was added.The mixture was heated to 399 k, the methanol was distilled off. After stirring at this temperature for 4 h,the reaction finished. The solvent was evaporated under reduced pressure. The product was purified by chromatography on silica. Crystals of hexanedioic acid dipyridin-4-ylmethyl ester were obtained by slow evaporation of a solution of ethyl acetation at room temperature(m.p. 359 k).

### Refinement

H atoms were positioned geometrically and refined as riding on their parent atoms [C—H distances are 0.93 Å (aromatic) and 0.97 Å (methylene) with *U*_iso_(H) = 1.2 *U*_eq_(C)].

### Crystal data

**Table:**

C_18_H_20_N_2_O_4_	*F*_000_ = 348
*M**_r_* = 328.36	*D*_x_ = 1.345 Mg m^−^^3^
Monoclinic, *P*2_1_/*c*	Mo *K*α radiation λ = 0.71073 Å
Hall symbol: -P 2ybc	Cell parameters from 2434 reflections
*a* = 9.1489 (18) Å	θ = 2.3–27.9º
*b* = 10.164 (2) Å	µ = 0.10 mm^−^^1^
*c* = 8.9206 (18) Å	*T* = 113 (2) K
β = 102.11 (3)º	Block, colorless
*V* = 811.1 (3) Å^3^	0.12 × 0.10 × 0.08 mm
*Z* = 2	

### Data collection

**Table:**

Rigaku Saturn diffractometer	1918 independent reflections
Radiation source: rotating anode	1288 reflections with *I* > 2σ(*I*)
Monochromator: confocal	*R*_int_ = 0.060
*T* = 113(2) K	θ_max_ = 27.9º
ω scans	θ_min_ = 2.3º
Absorption correction: multi-scan(CrystalClear; Rigaku, 2005)	*h* = −12→12
*T*_min_ = 0.979, *T*_max_ = 0.988	*k* = −13→13
9823 measured reflections	*l* = −11→11

### Refinement

**Table:**

Refinement on *F*^2^	Secondary atom site location: difference Fourier map
Least-squares matrix: full	Hydrogen site location: inferred from neighbouring sites
*R*[*F*^2^ > 2σ(*F*^2^)] = 0.041	H-atom parameters constrained
*wR*(*F*^2^) = 0.107	*w* = 1/[σ^2^(*F*_o_^2^) + (0.0564*P*)^2^] where *P* = (*F*_o_^2^ + 2*F*_c_^2^)/3
*S* = 0.98	(Δ/σ)_max_ < 0.001
1918 reflections	Δρ_max_ = 0.34 e Å^−^^3^
109 parameters	Δρ_min_ = −0.24 e Å^−^^3^
Primary atom site location: structure-invariant direct methods	Extinction correction: none

### Special details

**Table:**

Geometry. All esds (except the esd in the dihedral angle between two l.s. planes) are estimated using the full covariance matrix. The cell esds are taken into account individually in the estimation of esds in distances, angles and torsion angles; correlations between esds in cell parameters are only used when they are defined by crystal symmetry. An approximate (isotropic) treatment of cell esds is used for estimating esds involving l.s. planes.
Refinement. Refinement of *F*^2^ against ALL reflections. The weighted *R*-factor *wR* and goodness of fit *S* are based on *F*^2^, conventional *R*-factors *R* are based on *F*, with *F* set to zero for negative *F*^2^. The threshold expression of *F*^2^ > σ(*F*^2^) is used only for calculating *R*-factors(gt) *etc*. and is not relevant to the choice of reflections for refinement. *R*-factors based on *F*^2^ are statistically about twice as large as those based on *F*, and *R*- factors based on ALL data will be even larger.

### Fractional atomic coordinates and isotropic or equivalent isotropic displacement parameters (Å^2^)

**Table:**

	*x*	*y*	*z*	*U*_iso_*/*U*_eq_	
O1	0.70599 (9)	0.51214 (8)	0.11415 (9)	0.0198 (2)	
O2	0.64123 (11)	0.69241 (9)	0.22847 (10)	0.0307 (3)	
N1	0.90646 (13)	0.38939 (10)	−0.35175 (12)	0.0234 (3)	
C1	0.89598 (14)	0.58417 (13)	−0.20251 (14)	0.0219 (3)	
H1	0.9218	0.6722	−0.1854	0.026*	
C2	0.93751 (15)	0.51700 (13)	−0.32073 (15)	0.0226 (3)	
H2	0.9901	0.5625	−0.3828	0.027*	
C3	0.83041 (15)	0.32799 (13)	−0.25920 (15)	0.0250 (3)	
H3	0.8082	0.2394	−0.2773	0.030*	
C4	0.78264 (14)	0.38780 (13)	−0.13832 (14)	0.0221 (3)	
H4	0.7297	0.3404	−0.0781	0.026*	
C5	0.81535 (14)	0.51996 (12)	−0.10895 (13)	0.0184 (3)	
C6	0.76640 (15)	0.59800 (12)	0.01395 (14)	0.0206 (3)	
H6A	0.8509	0.6460	0.0727	0.025*	
H6B	0.6910	0.6614	−0.0323	0.025*	
C7	0.64740 (13)	0.57452 (13)	0.22139 (13)	0.0188 (3)	
C8	0.59372 (14)	0.47965 (12)	0.32611 (14)	0.0200 (3)	
H8A	0.6751	0.4212	0.3707	0.024*	
H8B	0.5141	0.4264	0.2669	0.024*	
C9	0.53658 (14)	0.54786 (11)	0.45402 (14)	0.0196 (3)	
H9A	0.6193	0.5912	0.5219	0.023*	
H9B	0.4647	0.6148	0.4102	0.023*	

### Atomic displacement parameters (Å^2^)

**Table:**

	*U*^11^	*U*^22^	*U*^33^	*U*^12^	*U*^13^	*U*^23^
O1	0.0269 (5)	0.0179 (5)	0.0184 (5)	0.0006 (4)	0.0133 (4)	0.0013 (4)
O2	0.0492 (7)	0.0193 (5)	0.0301 (6)	−0.0007 (4)	0.0230 (5)	−0.0018 (4)
N1	0.0283 (6)	0.0223 (6)	0.0218 (6)	0.0034 (5)	0.0102 (5)	−0.0005 (5)
C1	0.0242 (7)	0.0193 (7)	0.0236 (7)	0.0012 (5)	0.0081 (6)	0.0020 (6)
C2	0.0244 (7)	0.0263 (7)	0.0197 (7)	0.0016 (6)	0.0106 (6)	0.0040 (6)
C3	0.0288 (8)	0.0211 (7)	0.0263 (8)	−0.0004 (6)	0.0089 (6)	−0.0040 (6)
C4	0.0235 (7)	0.0249 (7)	0.0199 (7)	−0.0010 (5)	0.0093 (6)	0.0018 (6)
C5	0.0189 (6)	0.0216 (7)	0.0145 (6)	0.0016 (5)	0.0033 (5)	0.0012 (5)
C6	0.0283 (7)	0.0175 (7)	0.0191 (7)	−0.0018 (5)	0.0122 (6)	0.0023 (5)
C7	0.0199 (7)	0.0208 (7)	0.0168 (7)	0.0002 (5)	0.0062 (5)	−0.0028 (6)
C8	0.0240 (7)	0.0183 (6)	0.0202 (7)	−0.0005 (5)	0.0106 (5)	0.0010 (5)
C9	0.0220 (7)	0.0204 (7)	0.0187 (7)	−0.0001 (5)	0.0098 (6)	0.0005 (6)

### Geometric parameters (Å, °)

**Table:**

O1—C7	1.3489 (14)	C4—H4	0.9300
O1—C6	1.4398 (14)	C5—C6	1.4959 (17)
O2—C7	1.2019 (15)	C6—H6A	0.9700
N1—C3	1.3402 (16)	C6—H6B	0.9700
N1—C2	1.3441 (16)	C7—C8	1.4954 (17)
C1—C2	1.3751 (17)	C8—C9	1.5186 (17)
C1—C5	1.3874 (16)	C8—H8A	0.9700
C1—H1	0.9300	C8—H8B	0.9700
C2—H2	0.9300	C9—C9^i^	1.516 (2)
C3—C4	1.3860 (17)	C9—H9A	0.9700
C3—H3	0.9300	C9—H9B	0.9700
C4—C5	1.3891 (18)		
			
C7—O1—C6	114.63 (10)	C5—C6—H6A	109.6
C3—N1—C2	115.92 (11)	O1—C6—H6B	109.6
C2—C1—C5	119.68 (12)	C5—C6—H6B	109.6
C2—C1—H1	120.2	H6A—C6—H6B	108.1
C5—C1—H1	120.2	O2—C7—O1	122.40 (11)
N1—C2—C1	123.82 (12)	O2—C7—C8	125.79 (11)
N1—C2—H2	118.1	O1—C7—C8	111.80 (11)
C1—C2—H2	118.1	C7—C8—C9	112.64 (10)
N1—C3—C4	124.35 (12)	C7—C8—H8A	109.1
N1—C3—H3	117.8	C9—C8—H8A	109.1
C4—C3—H3	117.8	C7—C8—H8B	109.1
C3—C4—C5	118.70 (11)	C9—C8—H8B	109.1
C3—C4—H4	120.6	H8A—C8—H8B	107.8
C5—C4—H4	120.6	C9^i^—C9—C8	111.98 (12)
C1—C5—C4	117.52 (11)	C9^i^—C9—H9A	109.2
C1—C5—C6	118.04 (11)	C8—C9—H9A	109.2
C4—C5—C6	124.42 (11)	C9^i^—C9—H9B	109.2
O1—C6—C5	110.27 (10)	C8—C9—H9B	109.2
O1—C6—H6A	109.6	H9A—C9—H9B	107.9
			
C1—C5—C6—O1	−170.85 (11)	O1—C7—C8—C9	176.27 (10)
C6—O1—C7—C8	−177.17 (10)		

Symmetry codes: (i) −*x*+1, −*y*+1, −*z*+1.

### Hydrogen-bond geometry (Å, °)

**Table:**

*D*—H···*A*	*D*—H	H···*A*	*D*···*A*	*D*—H···*A*
C6—H6B···O2^ii^	0.97	2.56	3.3333 (17)	137

Symmetry codes: (ii) *x*, −*y*+3/2, *z*−1/2.
